# Sleep Disorders and Obesity in Saudi Populations Across the Lifespan: A Systematic Review

**DOI:** 10.3390/jcm14248709

**Published:** 2025-12-09

**Authors:** Amal H. Aljohani, Abdullah Heliel Aljohani, Khalid Mohammed Alshammari

**Affiliations:** 1 Pediatric Department, College of Medicine, Taibah University, Madinah 42353, Saudi Arabia; 2 Family Medicine, Madinah Health Cluster, Madinah 42313, Saudi Arabia; 3 Family Medicine, North Borders Health Cluster, Arar 91431, Saudi Arabia

**Keywords:** obesity, overweight, sleep disorders, Saudi Arabia

## Abstract

**Background:** Obesity and sleep issues are pressing health challenges that are closely tied together. Poor sleep can lead to weight gain, and in turn, obesity can make sleep problems worse. In Saudi Arabia, lifestyle habits—like having late-night meals, excessive screen time, and a lack of physical activity—might worsen these relationships at every age. **Objectives:** This review explores the relationship between obesity and sleep disorders across different stages of life in Saudi populations. Methods: We conducted a systematic review following the PRISMA 2020 guidelines. From December 2023 to March 2024, we searched PubMed, Scopus, Web of Science, and ScienceDirect for English-language observational studies related to obesity and sleep among Saudi populations. We evaluated the risk of bias using the ROBINS-I tool. Results: We included fourteen studies, involving 14,876 participants (49.8% male). The findings consistently indicated that obesity is linked to poor sleep quality, shorter sleep duration, insomnia, excessive daytime sleepiness, and breathing issues during sleep such as obstructive sleep apnea. Most studies showed a moderate risk of bias, mainly due to their cross-sectional designs and reliance on self-reported sleep data. **Conclusions:** Obesity and sleep disturbances are closely linked across all age groups in Saudi Arabia. Most studies reported associations between higher body weight and poorer sleep outcomes, including shorter duration, lower quality, and increased risk of sleep-disordered breathing. These findings underscore the need for age-specific public health strategies that encourage healthy sleep habits and lifestyle behaviors. **Limitations:** Variability in measurement methods across studies prevented us from conducting a meta-analysis. **Funding/Registration:** No funding; review not registered.

## 1. Introduction

Obesity is a complex and multifactorial disease [1]. About one-third of the world’s population now has a BMI of 25 kg/m^2^ or higher, indicating a significant increase in overweight and obesity rates over the past thirty years. BMI, calculated as body weight in kilograms divided by the square of height in meters (kg/m^2^), is the most widely used measure to assess obesity. Individuals with a BMI of 25 kg/m^2^ or greater are classified as overweight, while those with a BMI of 30 kg/m^2^ or higher are considered obese [2].

Obesity is a major global public health issue because of its increasing prevalence and strong links to various comorbidities such as cardiovascular disease, chronic respiratory conditions, type 2 diabetes, endocrine disorders, and multiple cancer types [1,3,4,5]. These obesity-related conditions increase healthcare costs and place a substantial economic and societal burden on communities. The primary drivers of the global obesity epidemic are modern dietary and lifestyle shifts, including increased consumption of high-calorie, nutrient-poor foods and decreased physical activity, which collectively create a persistent positive energy balance and weight gain [6,7].

Sleep disturbances (SDs) affect millions worldwide. Data from the National Health Interview Survey (NHIS) between 2004 and 2006 indicate that 70 million Americans experienced chronic sleep loss and sleep disruptions, which are strongly linked to poor health outcomes [8]. Adequate sleep is essential for maintaining overall health, supporting memory consolidation, tissue repair, metabolic regulation, and homeostatic balance. Proper immune and endocrine system functioning also relies heavily on sufficient sleep [8,9,10].

The National Sleep Foundation recommends 7–9 h of sleep for adults aged 18–64, 7–8 h for older adults (aged 65 and above), and more than 9 h for children under 14 [11]. Given the well-established association between sleep disorders and obesity, assessing sleep quality is crucial in managing patients with excess body weight [12,13].

Short sleep duration has been positively associated with obesity in both adolescents and adults; however, the mechanisms underlying this association may differ. Reduced sleep can lead to behavioral and hormonal changes that increase food intake, promote unhealthy dietary habits, and reduce physical activity, thereby contributing to weight gain [14,15]. Conversely, obesity itself can exacerbate sleep disturbances through comorbid conditions such as obstructive sleep apnea (OSA), functional gastrointestinal disorders (e.g., irritable bowel syndrome, functional dyspepsia), nocturia, asthma, and musculoskeletal pain [16].

This review aims to summarize the evidence regarding the link between obesity and sleep disturbances and to pinpoint factors specific to Saudi Arabia that contribute to these issues.

## 2. Methodology

This systematic review was conducted in accordance with the Preferred Reporting Items for Systematic Reviews and Meta-Analyses (PRISMA) guidelines (Table S1: PRISMA-checklist) [17]. Databases were searched from December 2023 to March 2024.

### 2.1. Search Strategy

To identify relevant literature, two reviewers independently performed ROBINS-I assessments; discrepancies were resolved by consensus. They screened the results and extracted data across four databases: PubMed, Scopus, Web of Science, and ScienceDirect. The search was restricted to English-language publications, and the unique indexing requirements of each database were considered.

The following keywords were transformed into PubMed MeSH terms and applied to locate relevant studies: “Obesity,” “Overweight,” “Sleep patterns,” “Sleep duration,” “Sleep disorders,” and “Saudi Arabia.” Boolean operators “OR” and “AND” were used to combine and refine results. Eligible publications included full-text, English-language articles involving human subjects and freely accessible data.

### 2.2. Selection Criteria

Inclusion criteria focused on studies that summarized data on the relationship between obesity and sleep disturbances and identified potential correlations. Only research conducted in Saudi Arabia was included, with no restrictions on participant age—studies involving human subjects, published in English, and freely accessible were considered.

Exclusion criteria included non-Saudi studies, non-English papers, review articles without primary data, and abstracts lacking full-text availability.

### 2.3. Data Extraction

The output from the search process was verified twice using Rayyan (QCRI) software (Rayyan, 2016; accessed March 2024) [18]. Researchers independently reviewed titles and abstracts according to inclusion and exclusion criteria. Any disagreements were resolved through discussion among reviewers.

For the included studies, data were collected using a predefined form that captured information on study titles, authors, publication year, study location, participant demographics, sleep assessment methods, and primary outcomes. Primary outcomes included body mass index (BMI), sleep duration, sleep quality, and the prevalence of sleep-related disorders, including obstructive sleep apnea (OSA). A separate sheet was used to record the risk-of-bias assessment. Other extracted data included study design and sample size, where available.

### 2.4. Strategy for Data Synthesis

A qualitative evaluation was performed by developing summary tables that integrated data from relevant studies. All age groups were included in the initial analysis. During synthesis, studies were subsequently summarized according to participant age (children, adolescents, adults) to highlight population-specific trends. Data synthesis focused on identifying patterns between sleep quality, duration, and obesity prevalence in Saudi populations.

A meta-analysis was not performed due to substantial heterogeneity across the included studies. This heterogeneity stemmed from several sources: differences in study designs (e.g., cross-sectional vs. retrospective), wide variation in participant age ranges (from young children to older adults), the use of various sleep assessment tools (e.g., PSG: polysomnography; PSQI: Pittsburgh Sleep Quality Index; ESS: Epworth Sleepiness Scale; ATLS: Arab Teens Lifestyle Study questionnaire; and self-reported questionnaires), and inconsistent definitions and measurements of both obesity and sleep disorders. Lifestyle factors, such as screen time, diet, and physical activity levels, also varied across studies, further complicating comparability. Given this variability, pooling data—and producing a forest plot—was methodologically inappropriate. Consequently, no sensitivity analyses or formal publication bias assessments were conducted. This approach aligns with PRISMA 2020 recommendations for conducting narrative synthesis when meta-analysis is not feasible.

### 2.5. Risk of Bias Assessment

The quality of included studies was evaluated using the ROBINS-I (Risk Of Bias In Non-randomized Studies of Interventions) tool [19]. We selected ROBINS-I because of its domain-based structure, which is appropriate for evaluating non-randomized studies. Although ROBINS-E was developed for studies of exposure, it was neither validated nor publicly available at the time we planned our review. The Newcastle–Ottawa Scale was also considered but deemed less suitable due to its limited domain coverage and its primary focus on cohort and case–control designs. The ROBINS-I assessment covered seven domains: confounding, participant selection, classification of exposures, deviations from intended interventions, missing data, outcome measurement, and selective reporting. Reporting bias was not formally assessed due to the small number of studies included. Certainty of evidence was not graded using GRADE because of substantial heterogeneity in study methodologies and outcomes.

## 3. Results

**Figure 1:**
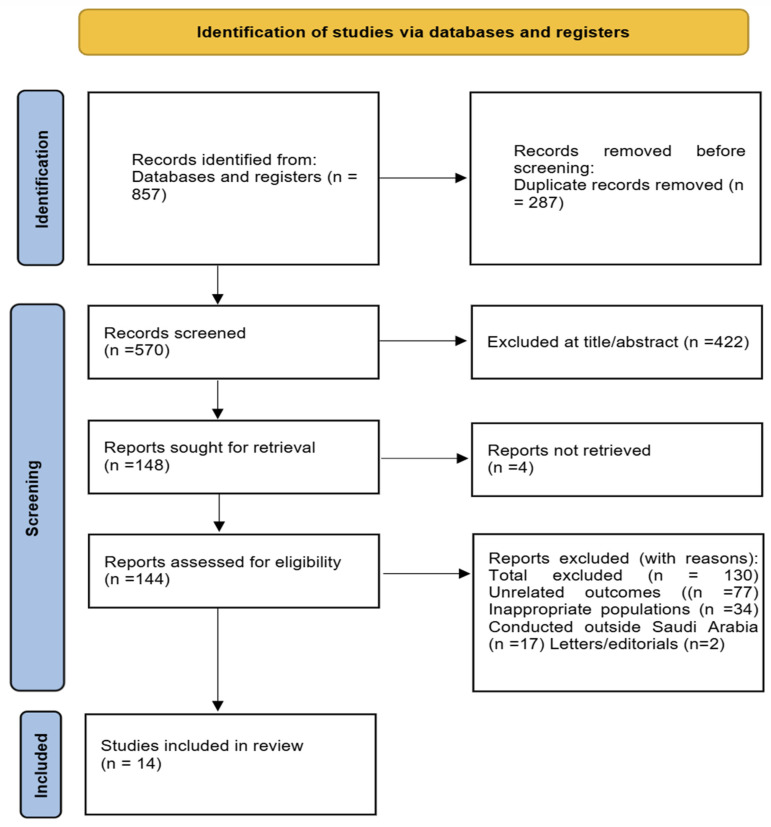
**PRISMA 2020 Flow Diagram for Study Selection.** Flowchart illustrating the identification, screening, eligibility, and inclusion of studies in the systematic review of sleep disorders and obesity across Saudi populations.

A total of 857 articles were identified through the systematic search. After removing 287 duplicates, 570 unique records remained for screening. Following title and abstract review, 422 studies were excluded for not meeting the inclusion criteria. Full-text retrieval was attempted for 148 reports; 144 full-text articles were successfully obtained and evaluated in detail, while four could not be retrieved. Among these, 77 were excluded due to unrelated outcomes, 34 due to inappropriate populations, 17 due to being conducted outside Saudi Arabia, and 2 were letters to the editor. Ultimately, 14 studies fulfilled all inclusion criteria and were included in this systematic review. A list of excluded full-text studies with reasons for exclusion (e.g., unrelated outcomes, wrong population, lack of full-text availability) is provided (Figure 1).

Together, these studies included 14,876 participants, of whom 7413 were male (49.8%). The majority adopted a cross-sectional design as illustrated in Table 1 [20,21,22,23,24,25,26,27,28,29,30], while two employed a retrospective approach [31,32]. Geographically, five studies were conducted in Riyadh [20,23,24,31,32], two in Jeddah [21,33], and the remaining studies across Hail [22], Madinah [25], Al-Khobar [26], Taif [27], Al-Hasa [28], Dammam [29], and Qassim [30].

Sample sizes ranged from fewer than 100 to nearly 3000 participants, encompassing diverse age groups, including children, adolescents, university students, and adults. Both genders were well represented. An overview of the study selection process is depicted in Figure 1, and a summary of the sociodemographic characteristics of the included studies is presented in Table 1.

Across the reviewed studies, obesity was consistently associated with multiple sleep disturbances, including poor sleep quality [30], insomnia and difficulty maintaining sleep [20], insufficient nighttime sleep [24,26,33], and excessive daytime sleepiness [27]. Sleep-disordered breathing, particularly obstructive sleep apnea and hypopnea, was markedly more prevalent and severe among individuals with obesity [21,25,31,32]. While we did not perform a formal GRADE assessment, we qualitatively judged the certainty of evidence based on study design, sample size, and measurement tools. For example, associations between obesity and sleep-disordered breathing—supported by studies using objective tools like PSG—were considered stronger. In contrast, findings related to sleep quality or insomnia, which relied on self-reported data, were judged to have lower confidence.

Among children, obesity emerged as a risk factor for sleep-disordered breathing [25,31,33], except for one pediatric study that did not find an independent association between obesity and short sleep duration [24]. Whereas in adults, severe obesity was linked to complications such as obesity hypoventilation syndrome [32]. In adolescents and young adults, lifestyle factors, including increased screen time, sedentary behavior, and unhealthy dietary patterns, intensify the association between obesity and sleep disorders [22,24,26,33]. Psychosocial factors, especially anxiety and stress, also served as mediators in this complex relationship [20].

Collectively, these findings highlight the complex interplay between obesity and sleep disorders in Saudi populations, encompassing biological, behavioral, and environmental factors. Clinical characteristics and sleep-related outcomes are detailed in Table 2. Most studies demonstrated a moderate risk of bias, mainly due to self-reported sleep data and cross-sectional designs. Across the reviewed studies, a consistent link emerged between obesity and sleep disorders, demonstrating how excess body weight negatively affects multiple aspects of sleep health. Poor sleep quality was commonly reported among obese individuals [28,30], while insomnia and difficulty maintaining sleep were observed in both adults and adolescents, suggesting a potential impact of obesity on sleep regulation mechanisms [20]. Among children and adolescents, short sleep duration was particularly prevalent and strongly associated with an increased risk of obesity [24,26,33]. In adults and university groups, frequent reports of excessive daytime sleepiness likely indicate poor sleep efficiency and disrupted circadian rhythms [27]. Additionally, sleep-disordered breathing issues, such as obstructive sleep apnea (OSA) and hypopnea, were notably more common and severe in individuals with higher body mass index (BMI) levels [21,25,31,32]. In addition to physiological factors, lifestyle behaviors such as excessive screen time, sedentary routines, and poor dietary choices further strengthen the link between obesity and sleep disorders. Moreover, psychosocial elements like anxiety and emotional stress played a role in sleep disturbances, highlighting the complex and reciprocal relationship between obesity and poor sleep patterns.

A meta-analysis was not performed due to substantial heterogeneity in study design, participant characteristics, and outcome measures. Variation in age groups, sleep-assessment tools, and lifestyle behaviors appeared to contribute most to heterogeneity among study findings. No formal sensitivity analyses were conducted. Reporting bias was not assessed because of the limited number of studies included. Overall certainty of evidence was considered low to moderate, reflecting the predominance of observational study designs and variability in outcome measures.

## 4. Discussion

This review demonstrates an association between obesity and sleep disorders across all age groups in Saudi Arabia, with both prevalence and severity shaped by age, sleep type, and lifestyle behaviors. As results showed, obesity was repeatedly linked to various sleep disorders [24,26,27,30,33]. Consistent with the findings of Bacaro et al. [34], who reported inconsistent associations between long sleep duration and obesity, the evidence presented here supports short sleep duration as a more reliable predictor of obesity risk in both children and adults. Extended sleep duration was not associated with an increased risk of obesity, partially aligning with prior meta-analyses [35,36].

In Saudi Arabia, distinctive lifestyle patterns—late-night eating, high-calorie evening snacks, and prolonged screen exposure (especially among adolescents)—may intensify the link between poor sleep and obesity. The consistent association between short sleep duration and higher BMI in the included studies suggests that interventions promoting sleep hygiene, stable routines, and behavioral change could help reduce obesity risk and its related health problems [22,24,26,33]. Although the associations observed align with global data, specific patterns appear to be particularly influenced by sociocultural and environmental factors unique to Saudi Arabia. For example, cultural norms often favor late-night social meals and evening activities; moreover, the hot desert climate tends to encourage indoor, sedentary lifestyles during the day, both of which may contribute to poor sleep and weight gain. These region-specific factors underscore the need for culturally tailored public health interventions that support healthier daily routines, offer accessible indoor physical activity options, and raise awareness of the impact of disrupted sleep and nighttime behaviors.

In the studies included, gender differences are a significant variable in the relationship between sleep and obesity. Several studies reported that males exhibited higher rates of obesity and associated sleep disturbances such as obstructive sleep apnea [21,31,32] as well as shorter sleep durations and poorer sleep hygiene [20,26]. Conversely, females were more likely to report poor sleep quality [20,28]. They had a higher prevalence of certain health conditions, such as anemia and vitamin D deficiency [23], which could indirectly affect sleep and weight. Cultural and behavioral factors, including reduced physical activity among females and unhealthy dietary habits among males, were also cited as contributing to these patterns [24,33]. These findings underscore the importance of incorporating gender-specific considerations into future research and targeted interventions in Saudi Arabia.

### 4.1. Physiological Evidence and Mechanisms

The connection between sleep and obesity involves hormonal, behavioral, and physiological factors. Sleep deprivation alters the balance of appetite-regulating hormones—lowering leptin and increasing ghrelin—which promotes increased hunger and caloric intake [37]. It also triggers behavioral changes such as greater screen time, late-night eating, and reduced physical activity, all of which contribute to weight gain [38,39]. In the Saudi context, cultural habits such as late-night high-calorie meals, extended evening screen use, and irregular sleep schedules may amplify these effects by disrupting circadian rhythms and worsening hormonal imbalances, particularly among adolescents and adults. Additionally, obesity itself increases the risk of sleep-related breathing issues like sleep-disordered breathing (SDB) and obstructive sleep apnea (OSA), which disrupt sleep and exacerbate metabolic problems. Several studies have elaborated the biological basis of this relationship [40]. Chronic sleep deprivation alters the balance of leptin and ghrelin, increasing appetite and caloric intake [38]; however, Capers et al. [38] noted heterogeneity across studies. Short sleep also promotes fatigue, reduces activity, and increases caloric intake [39,41]. Longitudinal evidence supports these findings: in adults aged 50–71 years, sleeping fewer than five to six hours per night was associated with a 40% higher risk of developing obesity over 7.5 years [42]. Similarly, children sleeping one to two hours less than recommended had substantially higher odds of being overweight or obese [43].

While the precise mechanisms remain under investigation [41], OSA remains a key clinical mediator. Defined by recurrent upper airway collapse during sleep, OSA causes oxygen desaturation and sleep fragmentation [44,45]. This review confirms that SDB, including OSA and hypopnea, is both prevalent and severe among obese individuals [21,25,31,32], with risk increasing tenfold in those with a BMI above 29 kg/m^2^ [46]. Even in the absence of OSA, obese individuals often exhibit prolonged sleep latency, reduced REM sleep, and poor sleep efficiency, suggesting that obesity alone may disrupt sleep through metabolic and inflammatory pathways [47].

### 4.2. Age-Specific Comparisons

Among children, 23 30% are at risk for SDB or short sleep duration. Obesity significantly contributes to SDB, although its direct impact on sleep duration lessens when behavioral factors are taken into account. Dabbour et al. [31] identified obesity as a predictor of SDB, especially hypopnea and OSA. Meanwhile, Al-Hazzaa et al. [24] reported that 71% of Saudi children sleep less than nine hours at night, attributing the insufficient sleep mainly to behavioral rather than metabolic factors. Similarly, Hassan et al. [25] confirmed a bidirectional link, indicating that obese children are up to five times more likely to develop SDB, with boys being more affected. These results highlight the importance of early interventions focusing on sleep hygiene, reducing screen time, and routine screening for sleep issues, as shown in Table 3.

In adolescents, a lack of sufficient sleep is strongly linked to overweight and obesity, with prevalence rates of 20 25% among school populations. Al-Hazzaa et al. [26] found that nearly one-third of Saudi adolescents sleep less than seven hours per night. Short sleep duration is significantly associated with overweight, especially among males. Kabel et al. [27] highlighted lifestyle, hormonal, and dietary factors as significant determinants of poor sleep, while academic stress and technology use further exacerbate deprivation. Adolescence, therefore, represents a critical developmental window for interventions that promote balanced routines, stress management, and proper sleep patterns.

In adults, both obesity prevalence and OSA severity are higher, especially among men, with short sleep increasing obesity risk by almost 50%. Poor sleep quality affects roughly half of Saudi adults, sustaining a reciprocal link between sleep disruption and weight gain. Studies in university and clinical samples [23,28,29,30] have shown that inadequate sleep is associated with higher BMI, weight gain, and unhealthy behaviors, such as sedentary behavior, poor diet, and prolonged screen use. Population-level surveys [21,33] revealed low awareness of OSA and other sleep disorders, highlighting urban risk factors such as high intake of sugary beverages and reduced sleep time. Clinical research [32] further identified obesity hypoventilation syndrome (OHS), especially in women, as a frequent complication of obesity and OSA, often co-occurring with hypertension and diabetes. These findings emphasize that chronic sleep disruption may act as both a behavioral and physiological cause of obesity and metabolic disease, requiring comprehensive interventions that combine sleep hygiene, stress management, and lifestyle changes.

**Figure 2:**
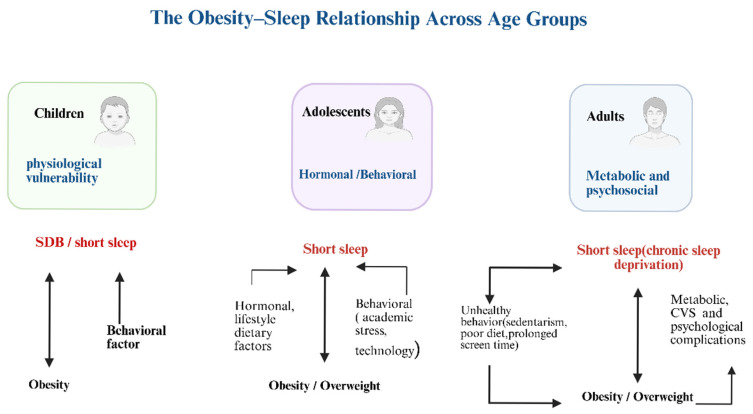
**Illustrates the obesity-sleep relationship across all age groups**. Created in BioRender. johani, A. (2025) https://BioRender.com/rz1irrg (accessed on 4 December 2025). SDB: Sleep-disordered breathing, CVS: Cardiovascular disease.

Across childhood, adolescence, and adulthood, the obesity sleep relationship transitions from physiological vulnerability to behavioral disruption, ultimately resulting in chronic systemic dysfunction (Figure 2). In children, SDB predominates; in adolescents, behavioral and hormonal influences drive short sleep and weight gain; in adults, chronic sleep deprivation contributes to metabolic, cardiovascular, and psychological complications. Across all life stages, inadequate sleep and obesity may reinforce one another in a self-perpetuating cycle [22,24,26,33]. Breaking this cycle requires age-specific strategies, including parental guidance and screen-time limits for children, lifestyle and stress interventions for adolescents, and metabolic and sleep health monitoring for adults. Saudi-specific habits such as late-night eating and prolonged screen use further aggravate this cycle. Promoting healthy sleep patterns may therefore serve as a core preventive measure for obesity and its comorbidities. Ultimately, this review suggests that obesity in Saudi Arabia may be closely associated with multiple sleep disorders. The underlying mechanisms involve hormonal imbalance, behavioral habits, and physiological changes that collectively worsen both sleep quality and metabolism.

While this review explored the association between sleep disorders and obesity across children, adolescents, and adults, it did not examine how these patterns track across the life course—such as whether sleep disturbances or obesity originating in childhood persist into adolescence or adulthood. Investigating these transitions requires prospective, long-term studies and remains an important area for future research. Furthermore, although the review highlights a consistent pattern of associations between obesity and various sleep disorders, these findings should be interpreted cautiously due to limitations in the underlying evidence. Most included studies were observational—primarily cross-sectional—and relied on self-reported measures of sleep and body weight. As a result, the overall certainty of evidence is low to moderate, and conclusions regarding causality or directionality remain tentative. Confounding, reporting bias, and methodological heterogeneity further limit the ability to draw definitive conclusions. Specifically, Saudi studies consistently report associations between poor sleep and obesity across age groups, but their cross-sectional design restricts causal inference [19,20,21]. In contrast, international longitudinal research supports a bidirectional relationship: short sleep increases the risk of obesity, and obesity worsens sleep disturbances, particularly sleep-disordered breathing [13,47]. These findings underscore the need for locally conducted prospective studies to clarify causal pathways and strengthen the Saudi evidence base.

## 5. Limitation

This review is limited by challenges in comparing sleep outcomes across studies due to varying definitions and measurement methods. Future research should account for confounders such as pubertal stage, circadian rhythm preference, and sleep duration. Several abstracts were excluded at the full-text stage, suggesting that this research area is still evolving. Most included studies were cross-sectional and relied on self-reported sleep, introducing potential recall and measurement bias. The most frequent sources of bias identified were confounding (e.g., unmeasured lifestyle factors) and outcome measurement. Additionally, restricting the search to peer-reviewed, English-language studies and excluding grey literature may have introduced selection and publication bias. The absence of a registered review protocol also limits reproducibility. While we followed PRISMA 2020 guidelines and reported our methods transparently, these constraints should be considered when interpreting the strength and generalizability of our conclusions.

## 6. Implications

Obesity and sleep disorders can be closely connected across all age groups, but their features vary. In children, moderate obesity can lead to SDB; adolescents exhibit a link between short sleep and obesity (~20–25%); and adults experience a higher prevalence of OSA and sleep issues related to obesity (~50% poor sleep). These results emphasize the need for age-specific strategies, such as sleep hygiene education, lifestyle changes, and obesity prevention, to lower sleep-related health risks in Saudi Arabia.

## 7. Conclusions

This review found consistent associations between poor sleep and obesity across age groups in Saudi Arabia, though causality cannot be confirmed due to predominantly cross-sectional data. Short sleep and irregular routines were linked to higher BMI in youth, while poor sleep quality and sleep-disordered breathing were common in obese adults. Sociocultural factors, such as late-night eating and sedentary lifestyles, may intensify these patterns. Age-specific, culturally tailored interventions are needed, along with longitudinal research to clarify causal pathways.

## Figures and Tables

**Table 1:** Sociodemographic characteristics of the included participants.

Study	Design	City	Participants (n)	Mean Age (Years)	Age Group	Males n (%)
Alhusseini et al., 2021 [20]	Cross-sectional	Riyadh	1113	18–45	Adults	327 (29.4)
Alshehri et al., 2019 [21]	Cross-sectional	Jeddah	803	44.9 ± 15.9	Adults	454 (56.3)
Abedelmalek et al., 2022 [22]	Cross-sectional	Hail	1598	12–15	Children	935 (55.8)
Alodhayani et al., 2017 [23]	Cross-sectional	Riyadh	408	20.7 ± 2.3	Young Adults	224 (54.9)
Dabbour, 2020 [31]	Retrospective	Riyadh	51	10.3 ± 2.3	Children	26 (51.0)
Al-Hazzaa et al., 2019 [24]	Cross-sectional	Riyadh	1051	9.2 ± 1.7	Children	523 (49.8)
Alsulami et al., 2023 [33]	Cross-sectional	Jeddah	659	18–30	Young Adults	313 (47.4)
Hassan et al., 2021 [25]	Cross-sectional	Madinah	1528	9.7 ± 1.4	Children	842 (53.3)
BaHammam, 2015 [32]	Retrospective	Riyadh	1693	47.7 ± 14.0	Adults	1076 (63.6)
Al-Hazzaa et al., 2012 [26]	Cross-sectional	Al-Khobar, Jeddah, Riyadh	2868	16.6 ± 1.1	Adolescents	1379 (48.1)
Kabel et al., 2018 [27]	Cross-sectional	Taif	1066	18–25	Adolescents and young adults	487 (45.7)
Al-Otaibi, 2016 [28]	Cross-sectional	Al-Hasa	233	22.4 ± 9.6	Young Adults	0
Rafique, 2023 [29]	Cross-sectional	Dammam	1724	16–22	Adolescents/Young Adults	768 (44.5)
Mohieldein, 2020 [30]	Cross-sectional	Qassim	81	18–46	Adults	59 (72.8)

**Table 2:** Clinical characteristics, sleep assessment methods, and obesity-related outcomes.

Study	Population	Sleep Assessment	Key Outcomes	Evidence Strength *	ROBINS-I Bias Rating *
Alhusseini et al., 2021 [20]	General population	** PSQI (Subjective)	Obesity, insomnia, and anxiety interrelate, worsening sleep and promoting emotional eating; poor sleep linked to obesity.	Moderate	Moderate
Alshehri et al., 2019 [21]	General population	PSG (Objective)	Obese individuals, especially men, show higher prevalence and severity of OSA; Class III obesity linked to severe OSA.	High	Low
Abedelmalek et al., 2022 [22]	Adolescents	ATLS (Subjective)	High obesity and overweight rates associated with diet, sleep, and activity; lifestyle modification recommended.	Moderate	Moderate
Alodhayani et al., 2017 [23]	Medical students	ESS (Subjective)	Sleep disruption associated with weight gain; sedentary reading and lying down post-lunch increase obesity risk.	Moderate	Moderate
Dabbour, 2020 [31]	Children	PSG (Objective)	Obesity is a risk factor for SDB; hypopnea most common, followed by OSA; BMI not correlated with OSA severity.	Moderate	Moderate
Al-Hazzaa et al., 2019 [24]	Children (6–13 yrs)	ATLS (Subjective)	Short sleep linked to male sex, low paternal education, infrequent breakfast, and long screen time; obesity not significant after adjustment.	High	Low
Alsulami et al., 2023 [33]	University students	ATLS (Subjective)	Obesity more prevalent in males (24%); predictors include male sex, short sleep, smoking, sugary drinks.	High	Low
Hassan et al., 2021 [25]	Children	PSQ (Subjective)	~23% at risk for SDB; strong correlation between obesity/overweight and SDB risk.	Moderate	Moderate
BaHammam, 2015 [32]	General population	PSG (Objective)	OSA co-occurs with obesity hypoventilation syndrome; predictors include bicarbonate levels and oxygen saturation time.	High	Low
Al-Hazzaa et al., 2012 [26]	Adolescents (15–19 yrs)	ATLS (Subjective)	Short sleep duration is associated with overweight and obesity among teenagers.	Moderate	Moderate
Kabel et al., 2018 [27]	University students	ESS (Subjective)	Sleep disturbances and EDS are linked to anemia, obesity, glucose intolerance, and high altitude.	Moderate	Moderate
Al-Otaibi, 2016 [28]	General population	PSQI (Subjective)	Poor sleep associated with physical inactivity, high waist circumference, and obesity.	Moderate	Moderate
Rafique, 2023 [29]	General population	Online questionnaire (Subjective)	Short sleep duration is linked to higher BMI; sleep deprivation is an independent risk factor for obesity (OR 1.47).	High	Low
Mohieldein, 2020 [30]	General population	Self-reported (Subjective)	7–8 h of sleep may reduce obesity risk; healthy sleep promotes better lifestyle choices.	Moderate	Moderate

* Evidence Strength is a qualitative judgment based on overall study design, sample size, objectivity of sleep assessment (e.g., PSG vs. self-reported), and alignment with review outcomes. * ROBINS-I Bias Rating reflects formal risk-of-bias assessments per the ROBINS-I tool across seven domains (confounding, selection, classification, etc.). ** PSQI—Pittsburgh Sleep Quality Index, PSG—Polysomnography, ESS—Epworth Sleepiness Scale, ATLS—Arab Teens Lifestyle Study questionnaire, PSQ—Pediatric Sleep Questionnaire, SDB—Sleep-Disordered Breathing, OSA—Obstructive Sleep Apnea, EDS—Excessive Daytime Sleepiness.

**Table 3:** Comparative summary of sleep–obesity associations across age groups in the included studies.

Category	Children	Adolescents	Adults
Main Focus	Physiological effects of obesity on sleep, especially sleep-disordered breathing (SDB)	Behavioral and hormonal influences on sleep duration and weight status	Lifestyle, psychological stress, and chronic health outcomes linked to sleep and obesity
Common Sleep Problems	SDB, habitual snoring, short sleep duration	Irregular sleep schedules, poor sleep hygiene, delayed sleep phase	Insomnia, poor sleep quality, obstructive sleep apnea (OSA), obesity hypoventilation syndrome (OHS)
Relationship to Obesity	Obesity increases SDB risk, but not always directly tied to short sleep	Short sleep duration strongly associated with overweight and obesity	Strong bidirectional association; poor sleep predicts higher BMI, weight gain, and metabolic dysfunction
Contributing Factors	High screen time, inconsistent sleep routines, family habits	Hormonal fluctuations, late-night device use, academic and social pressures	Sedentary behavior, stress, caffeine intake, unhealthy diet, long work hours, prolonged screen use
Gender Differences	Boys more prone to SDB	Males exhibit higher obesity rates and shorter sleep	Women more affected by OHS; stress and lifestyle contribute across genders
Health Implications	Respiratory issues, fatigue, reduced cognitive performance	Weight gain, mood disturbances, and decreased academic achievement	Hypertension, diabetes, metabolic syndrome, and depression
Recommended Interventions	Parental oversight, screen-time regulation, early SDB screening	Sleep education, stress reduction, promoting consistent routines	Workplace wellness programs, stress management, sleep hygiene promotion
Overall Insight	Sleep–obesity link primarily driven by physiological mechanisms	Behavioral and hormonal disruptions reinforce obesity risk	Complex metabolic and psychosocial feedback loops sustain obesity and poor sleep

## Data Availability

The data that support the findings of this study are available from the authors upon request.

## Supplementary Materials

The following are available online at https://www.mdpi.com/article/10.3390/jcm14248709/s1, Table S1: PRISMA-checklist.
